# Domain swapping reveals that the N-terminal domain of the sensor kinase KdpD in *Escherichia coli *is important for signaling

**DOI:** 10.1186/1471-2180-9-133

**Published:** 2009-07-09

**Authors:** Ralf Heermann, Marie-Luise Lippert, Kirsten Jung

**Affiliations:** 1 Ludwig-Maximilians-Universität München, Biozentrum, Bereich Mikrobiologie, Großhaderner Str. 2-4, D-82152 Martinsried, Germany; 2 Munich Center of integrated Protein Science CiPSM, München, Germany; 3 Philipps-Universität Marburg, Fachbereich Biologie, Mikrobiologie, Karl-von-Frisch-Str. 8, D-35043 Marburg, Germany

## Abstract

**Background:**

The KdpD/KdpE two-component system of *Escherichia coli *regulates expression of the *kdpFABC *operon encoding the high affinity K^+ ^transport system KdpFABC. The input domain of KdpD comprises a domain that belongs to the family of universal stress proteins (Usp). It has been previously demonstrated that UspC binds to this domain, resulting in KdpD/KdpE scaffolding under salt stress. However the mechanistic significance of this domain for signaling remains unclear. Here, we employed a "domain swapping" approach to replace the KdpD-Usp domain with four homologous domains or with the six soluble Usp proteins of *E. coli*.

**Results:**

Full response to salt stress was only achieved with a chimera that contains UspC, probably due to unaffected scaffolding of the KdpD/KdpE signaling cascade by soluble UspC. Unexpectedly, chimeras containing either UspF or UspG not only prevented *kdpFABC *expression under salt stress but also under K^+ ^limiting conditions, although these hybrid proteins exhibited kinase and phosphotransferase activities *in vitro*. These are the first KdpD derivatives that do not respond to K^+ ^limitation due to alterations in the N-terminal domain. Analysis of the KdpD-Usp tertiary structure revealed that this domain has a net positively charged surface, while UspF and UspG are characterized by net negative surface charges.

**Conclusion:**

The Usp domain within KdpD not only functions as a binding surface for the scaffold UspC, but it is also important for KdpD signaling. We propose that KdpD sensing/signaling involves alterations of electrostatic interactions between the large N- and C-terminal cytoplasmic domains.

## Background

K^+ ^plays an important role in turgor maintenance in bacteria [1]. KdpFABC is a high affinity K^+ ^uptake system that serves as an emergency system to scavenge K^+ ^when other transporters cannot sustain the cellular requirement for K^+^. The corresponding *kdpFABC *operon is under control of the two-component system KdpD/KdpE, which induces *kdpFABC *expression under K^+ ^limiting conditions or under osmotic stress imposed by a salt [2,3]. Upon stimulus perception, KdpD undergoes autophosphorylation and subsequently, the phosphoryl group is transferred to the cytoplasmic response regulator KdpE [4]. Phosphorylated KdpE exhibits increased affinity for a 23-base pair sequence upstream of the canonical -35 and -10 regions of the *kdpFABC *promoter and triggers *kdpFABC *expression [5]. The enzymatic activities of purified KdpD and KdpE were determined *in vitro *[4]. All data known thus far indicate that KdpD does not sense a single specific parameter, but integrates the information of intracellular parameters imposed by K^+ ^limitation or salt stress. The current working model proposes that KdpD perceives alterations in the intracellular K^+ ^concentration, the ionic strength, and the ATP concentration as stimuli [6].

KdpD consists of a characteristic C-terminal transmitter domain, which is fused via a small linker region to the large N-terminal input domain. Several regions of the input domain have been identified as important for stimulus perception and integration. The four transmembrane domains (TM1-TM4) anchor the sensor kinase in the cytoplasmic membrane and separate the two large cytoplasmic domains from each other [7,8]. The transmembrane helices TM2 and TM3 function as a type of clip and are responsible for the correct positioning of the large cytoplasmic domains relative to each other [8]. We have previously shown a direct interaction between these KdpD cytoplasmic domains [9]. The α-helix of TM4 extends from the membrane into the cytoplasm and encompasses a cluster of positively charged amino acids (R503-R511) that are mainly involved in stimulus perception, and has therefore been proposed as a K^+ ^binding site by Altendorf and coworkers [10,11]. This hypothesis is in accord with the finding that amino acid replacements resulting in K^+^-independent *kdpFABC *expression are located within TM4 and the adjacent region [11-13]. It was previously shown that the cluster of positively charged amino acids is important for modulation of the kinase and phosphatase activity, because individual replacements of these amino acids resulted in KdpD derivatives with either enhanced kinase and reduced phosphatase activity, or enhanced phosphatase and reduced kinase activity [10]. Furthermore, a KdpD derivative lacking the cytoplasmic N-terminal region and the first two transmembrane domains of KdpD were able to respond to K^+ ^limitation, which supports the assumption that the K^+ ^binding site is located within this region [14].

**Figure 1:**
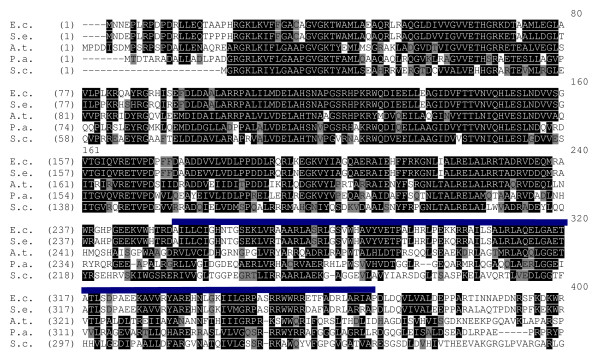
**Sequence alignment of the N-terminal domain of KdpD (KdpD/1-395) comprising the Usp-domain, marked by the blue line**. The alignment was created and identities/similarities were determined using VectorNTI AlignX. E.c. (*Escherichia coli*), S.e. (*Salmonella enterica *serotype Typhimurium), A.t. (*Agrobacterium tumefaciens*), P.a. (*Pseudomonas aeruginosa*), S.c. (*Streptomyces coelicolor*).

**Figure 2:**
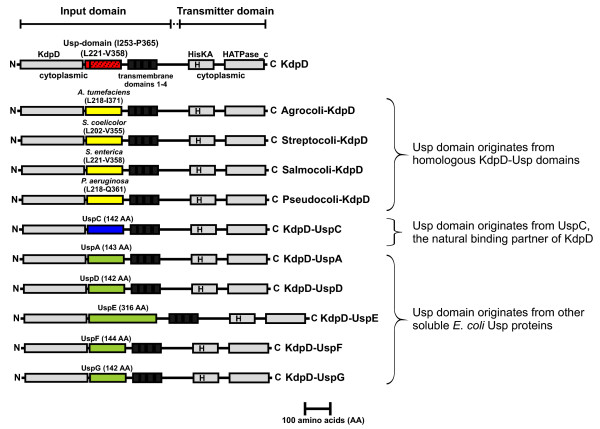
**Schematic presentation of the domain structure of the sensor kinase KdpD and the KdpD-Usp chimeras investigated in this study**. The model is based on hydropathy plot analysis, studies with *lacZ*/*phoA *fusions [7], and use of the conserved domain architecture retrieval tool (CDART) [26]. KdpD contains the conserved domains of histidine kinases: HATPase_c (Histidine kinase-like ATPases; Histidine kinase-, DNA gyrase B-, phytochrome-like ATPases, SMART00387) and HisKA (His Kinase A phosphoacceptor domain; dimerization and phosphoacceptor domain of histidine kinases, SMART00388). Within the input domain, the location of the highly conserved KdpD domain (pfam02702, presented in grey) and the Usp domain USP-OKCHK (cd01987, pfam00582, highlighted by dots) are shown. Amino acids comprising the KdpD-Usp domain (red box) were replaced with the corresponding amino acid sequences of four homologous KdpD-Usp domains (yellow boxes) or with the soluble Usp proteins (green boxes) of *E. coli*. UspC is the native binding partner of KdpD; the replacement of KdpD-Usp with UspC is marked by a blue box. The first and last amino acid of the homologous KdpD-Usp domains as well as the number of replaced amino acids comprising the respective soluble Usp protein are indicated above the Usp-domains of the chimeras.

The role of the KdpD N-terminal input domain large cytoplasmic region (M1-W395, Fig. 1) for sensing and signal transduction has been a mystery for a long time. Altendorf and coworkers found that truncations within the N-terminal domain resulted in functional KdpD protein *in vitro *[15]. Later, a sequence motif was identified within this domain that is very similar to the classical "Walker A" motif [16]. Truncations that encompass this motif (R12-D228, R12-W395) result in deregulated phosphatase activity [16]. Since ATP-binding within this region is known to be involved in modulation of the phosphatase activity, ATP may function as an intracellular stimulus that is sensed by KdpD under osmotic stress [9,16]. This is in accord with the finding that the intracellular ATP concentration increases significantly upon an osmotic upshift [17]. A truncated version of KdpD comprising only the N-terminal cytoplasmic domain (KdpD/1-395) caused constitutive expression of *kdpFABC in vivo*, revealing a stabilizing function of the N-terminal domain of KdpD in complex with phosphorylated KdpE and the corresponding DNA binding site [8]. This hydrophilic N-terminal domain contains two subdomains: a KdpD domain (pfam02702) and a Usp domain (cd01987) (Figs. 1 and 2). The Usp domain within KdpD (I253-P365) shares similarities to the Usp proteins of the UspA subfamily [18]. The KdpD-Usp domain binds the universal stress protein UspC [19]. It has been puzzling how KdpD is activated under salt stress when K^+ ^accumulates [20], although the kinase activity is inhibited by K^+ ^[21]. Recent data indicate that UspC scaffolds the KdpD/KdpE signaling cascade under salt stress by stabilizing the KdpD/KdpE~P/DNA complex [19]. This is in accord with the earlier finding according to which cells producing a truncated KdpD lacking the Usp domain exhibit reduced *kdpFABC *expression under salt stress [15].

The Usp superfamily encompasses an ancient and conserved group of proteins that are found in bacteria, archaea, fungi, flies, and plants (see [22] for review). Usp-containing organisms are usually equipped with several copies of *usp *genes. The *usp *genes encode either small Usp proteins (one Usp domain), larger versions with two Usp domains in tandem, or Usp domains integrated in multi-domain proteins [18]. *E. coli *contains six Usp proteins that can be divided into two subfamilies on the basis of sequence similarities [23]. Three of these proteins belong to the UspA subfamily (UspA, UspC, and UspD), and two to the UspFG subfamily (UspF and UspG). The UspE protein is a tandem-like protein consisting of two Usp domains. The UspE domain1 is more related to the UspA sub-family, whereas the domain2 is closer related to the UspFG sub-family. The intracellular copy number of UspA, UspC, UspD, and UspE increases upon stress conditions such as starvation, moderate heat stress, oxidative stress, and osmotic stress [23]. UspG is induced under osmotic stress and has recently been shown to undergo autophosphorylation and autoadenylation [24]. However, the exact functions of these small proteins are unclear.

The degree of similarity of the Usp domain within KdpD (Fig. 1) varies among all known KdpD sequences. To elucidate the role of the Usp domain in KdpD for signaling, we used a "domain swapping" approach, wherein the *E. coli *KdpD-Usp domain was replaced with homologous domains or the six *E. coli *Usp proteins. These KdpD chimeras were characterized *in vivo *as well as *in vitro*.

## Results

### "Domain swapping" of the Usp domain within KdpD

The N-terminal region of the cytoplasmic input domain containing the KdpD domain (pfam02702) is highly conserved [25], whereas the C-terminal region containing the Usp-domain (cd01987) (I253-P365) is less conserved (Fig. 1). The KdpD-Usp domain of other bacteria, for example *Agrobacterium tumefaciens *(KdpD/R249-D372), *Streptomyces coelicolor *(KdpD/R233-I354), *Salmonella enterica *serotype Typhimurium (KdpD/I253-P365), and *Pseudomonas aeruginosa *(KdpD/R248-R358) are characterized by different degrees of identity and similarity. The highest degree of sequence identity has the KdpD-Usp domain of *S. enterica *serotype Typhimurium compared to the corresponding *E. coli *domain (86% identity, 89% similarity). The other KdpD-Usp domains are less conserved (*A. tumefaciens*: 30% identity, 45% similarity; *P. aeruginosa*: 28% identity, 43% similarity; *S. coelicolor*: 25% identity, 42% similarity). The KdpD-Usp domain belongs to the UspA subfamily. Despite the lack of amino acid sequence identity, proteins of this (sub)family (UspA, UspC and UspD) are predicted to have a homologous tertiary structure which consists of four to five central β-sheets surrounded by four a-helices [19,22]. To examine the specifics of the KdpD-Usp domain and its importance in KdpD signaling, we replaced amino acids L221-V358 of *E. coli *KdpD with the homologous KdpD-Usp domains of *A. tumefaciens *(L218-I371), *S. enterica *serotype Typhimurium (L221-V358), *S. coelicolor *(L202-V355), and *P. aeruginosa *(L218-Q361) as described in *Methods*, and designated the chimeras Agrocoli-KdpD, Salmocoli-KdpD, Streptocoli-KdpD, and Pseudocoli-KdpD (Fig. 2) [26]. Furthermore, we exchanged the KdpD-Usp domain of *E. coli *with the six soluble Usp protein sequences of *E. coli*, yielding the chimeras KdpD-UspA, KdpD-UspC, KdpD-UspD, KdpD-UspE, KdpD-UspF, and KdpD-UspG (Fig. 2). Since the predicted tertiary structure of these domains is rather similar, we expected no major effects on the overall tertiary structure of KdpD.

**Figure 3:**
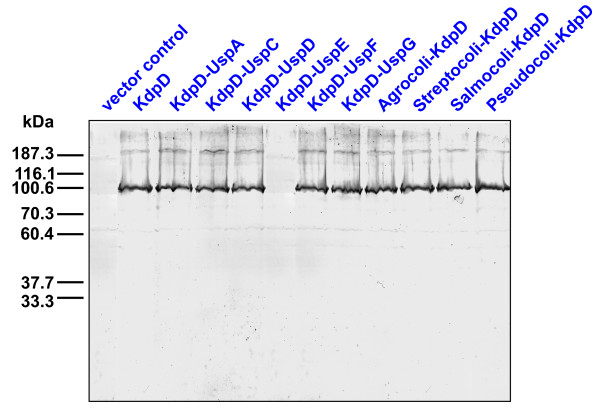
**Detection of the KdpD-Usp chimeras**. *E. coli *strain LMG194 was transformed with the pBD plasmids encoding the different KdpD-Usp chimeras or the empty vector pBAD18 (vector control). Overproduction of the indicated proteins was achieved by addition of 0.2% (w/v) arabinose. Cells were harvested in the mid-logarithmic growth phase, disrupted by addition of SDS-sample buffer [36], and subjected to a 10% SDS-gel. The KdpD chimeras were detected by immunoblotting with polyclonal antibodies against KdpD.

Plasmid pBD and the corresponding derivatives encoding the KdpD-Usp chimeras were introduced into *E. coli *LMG194, and protein overproduction was induced by arabinose. As shown in Fig. 3, all hybrid proteins were produced in nearly the same concentration, except KdpD-UspE. Even when this construct was put under control of the strong *tac *promoter (*E. coli *TKR2000/pPV5-3/UspE), we were not able to detect KdpD-UspE. UspE contains two Usp domains in tandem. Therefore, it is conceivable that insertion of this protein causes major structural changes hindering membrane insertion. For that reason KdpD-UspE was not further characterized *in vivo *or *in vitro*.

### The response of KdpD-Usp chimeras to salt stress

**Figure 4:**
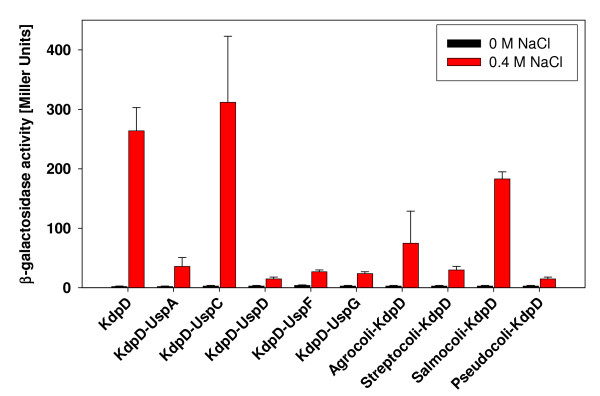
**The response of different KdpD-Usp chimeras to salt stress**. Plasmids expressing the indicated proteins were transformed in *E. coli *HAK006 as reporter strain. Cells were grown in minimal medium containing 5 mM K^+ ^with or without 0.4 M sodium chloride. Cells were harvested in the mid-logarithmic growth phase, and β-galactosidase activity was determined, given in Miller Units [39]. The data are average values obtained from at least three independent experiments, and error bars represent standard deviations.

UspC has been identified as a scaffolding protein for the KdpD/KdpE signaling cascade under salt stress [19]. The different KdpD chimeras were tested for their functionality *in vivo*. For this purpose, we used the *E. coli *strain HAK006 that carries a fusion of the upstream region of the *kdpFABC *operon with a promoterless *lacZ *gene as a reporter strain [12,16]. Since the copy number of regulatory proteins is very critical in signal transduction, *E. coli *HAK006 was transformed with plasmid pBD and its derivatives, encoding the KdpD-Usp chimeras under control of the arabinose promoter. When cells are grown in the absence of the inducer arabinose and in the presence of the repressor glucose, the small amount of KdpD proteins produced is optimal to complement a *kdpD *null strain [16]. Cells harboring these pBD derivatives were grown in minimal medium of higher osmolarity imposed by the addition of 0.4 M NaCl, and β-galactosidase activities were determined as a measure of *kdpFABC *expression. KdpD-UspC, Salmocoli-KdpD and Agrocoli-KdpD were able to induce *kdpFABC *expression 20 to150-fold, respectively, in presence of salt stress compared to no stress (Fig. 4). The highest induction level was produced by KdpD-UspC (150-fold induction). Cells producing Salmocoli-KdpD and Agrocoli-KdpD responded to salt stress, however the induction level was lower (20 to 60-fold induction) compared to cells producing wild-type KdpD (130-fold induction). In contrast, KdpD-UspA, KdpD-UspD, KdpD-UspF, KdpD-UspG, Streptocoli-KdpD, and Pseudocoli-KdpD were unable to sense an increased osmolarity.

Usp proteins form homodimers and oligomers, thus it is conceivable that UspC interacts with KdpD-UspC and thereby facilitates scaffolding. Although the *Salmonella *KdpD-Usp domain has the highest degree of similarity to the *E. coli *KdpD-Usp-domain, scaffolding by UspC seemed to be abolished. The induction level supported by this chimera was comparable to wild-type KdpD in a Δ*uspC *mutant [19]. Scaffolding might also be prevented in Agrocoli-KdpD. These data underline the importance of the KdpD-Usp domain for scaffolding the KdpD/KdpE signaling cascade under salt stress. The negative results obtained for all other KdpD chimeras might be explained by steric hindrance of the protein dynamics due to binding of other Usp proteins, major structural changes, or altered enzymatic activities.

### The response of KdpD-Usp chimeras towards K^+ ^limitation

**Figure 5:**
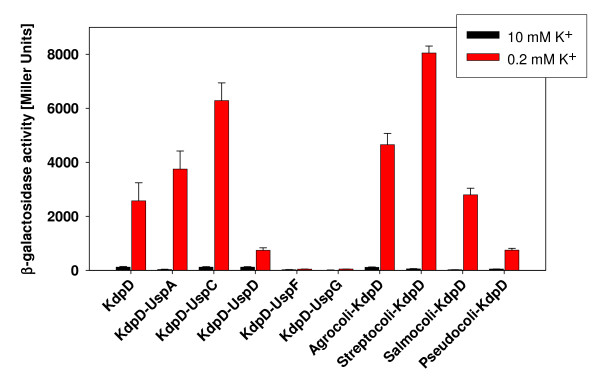
**The response of different KdpD-Usp chimeras to K^+ ^limitation**. Plasmids expressing the indicated proteins were transformed in *E. coli *HAK006 as reporter strain. Cells were grown in minimal media containing different K^+ ^concentrations (10 mM and 0.2 mM) to the mid-exponential phase, β-galactosidase activity was determined, and is given in Miller Units [39]. The data are average values obtained from at least three independent experiments, and error bars represent standard deviations.

All KdpD derivatives with altered osmosensing properties characterized thus far [8,10,12] were able to respond to K^+ ^limitation. To test the response toward K^+ ^limitation, cells producing the KdpD-Usp chimeras were grown in minimal media containing different K^+ ^concentrations. In wild-type cells, *kdpFABC *expression is repressed when cells are grown in medium that contains 10 mM K^+^, and induced under K^+ ^limiting conditions (0.2 mM K^+^) (Fig. 5). As shown earlier [19,27,28], the Kdp system is induced under K^+ ^limitation to a much higher level than in response to salt stress. None of the KdpD-Usp chimeras induced *kdpFABC *expression at a high K^+ ^concentration. As expected from the salt stress study, cells producing KdpD-UspC, Streptocoli-KdpD, or Agrocoli-KdpD induced *kdpFABC *expression similar to wild-type KdpD. Moreover, KdpD-UspA, KdpD-UspD and Pseudocoli-KdpD were able to respond to K^+ ^limitation, although the β-galactosidase activities were significantly reduced in Pseudocoli-KdpD and KdpD-UspD. Cells producing these chimeras were exposed to even more severe K^+ ^limitation (0.1 mM), and *kdpFABC *expression levels increased to wild-type levels, indicating that these two chimeras retain the ability to sense K^+ ^limitation (data not shown). Unexpectedly, KdpD-UspF and KdpD-UspG were unable to induce *kdpFABC *expression under all conditions tested ([K^+^] = 0.1 – 115 mM, data not shown). These are the first two KdpD derivatives with alterations in the N-terminal domain that completely prevent *kdpFABC *expression. These results reveal that the KdpD-Usp domain is not only a binding partner for UspC but is somehow involved in KdpD/KdpE signaling.

### The enzymatic activities of the KdpD-Usp chimeras *in vitro*

**Figure 6:**
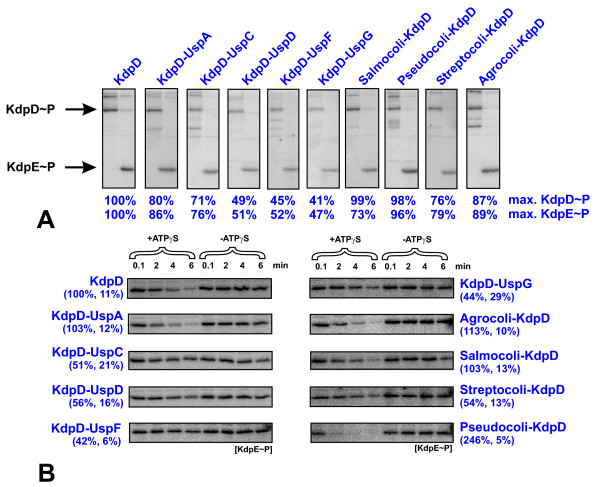
***In vitro *activities of the KdpD-Usp chimeras**. KdpD-autokinase and KdpE-phosphotransferase activities (**A**) as well as KdpE-phosphatase activities (**B**) were determined as described in *Methods*. Data are presented as percentages of maximal accumulation of KdpD~P or KdpE~P (after 3 min, kinase as well as phosphotransferase activity) (**A**), respectively, or as percentages of the dephosphorylation initial rates relative to wild-type KdpD (+/- ATPγS) (**B**). For wild-type KdpD (100% values), the autophosphorylation activity of KdpD was determined with 14 pmol min^-1 ^mg^-1 ^protein. The phosphatase activity was determined with 2.1 pmol min^-1 ^mg^-1 ^protein. The autoradiographs represent a typical result from three independently performed experiments, whereas the values represent the averaged results of these three independent measurements.

To test whether the sensing capabilities of the KdpD-Usp chimeras were related to altered enzymatic activities, we determined the activities of autokinase-, KdpE-phosphotransferase-, and KdpE-phosphatase for each chimera (see *Methods *for details). All KdpD-Usp chimeras exhibited kinase and KdpE-phosphotransferase activity (Fig. 6A). KdpD has an ATP-dependent phosphatase activity, which is modulated upon binding of ATP to the N-terminal KdpD-domain [9,16]. The ATP-dependency of the phosphatase activity was not changed in any of the KdpD-Usp chimeras, because significant dephosphorylation could only be observed in the presence of ATP (Fig. 6B). Despite detection of enzymatic activities for all chimeras, the ratio between kinase-phosphotransferase to phosphatase activities is important for the corresponding output (Table 1). The ratios for Salmocoli-KdpD, Agrocoli-KdpD and KdpD-UspA, KdpD-UspD, KdpD-UspF, KdpD-UspG were comparable to wild-type KdpD (deviation less than 20%). In KdpD-UspC and Streptocoli-KdpD, these ratios were shifted toward the kinase-phosphotransferase activity, resulting in higher levels of phosphorylated KdpE. The enhanced *kdpFABC *expression mediated by KdpD-UspC and Streptocoli-KdpD under K^+ ^limitation can therefore be explained by decreased phosphatase activities (Fig. 6B). Pseudocoli-KdpD was characterized by a ratio that was drastically shifted to the phosphatase activity, resulting in less phosphorylated KdpE. This result might explain the low induction potential of this chimera in response to K^+ ^limitation and salt stress. Remarkably, KdpD-UspF and KdpD-UspG were characterized by decreased phosphatase activities.

**Table 1:** Kinase-phosphotransferase to phosphatase ratios of the KdpD chimeras.

Chimera	Kinase-phosphotransferase to phosphatase ratio
KdpD	1.00
KdpD-UspA	0.81
KdpD-UspC	1.44
KdpD-UspD	0.89
KdpD-UspF	1.15
KdpD-UspG	1.00
Agrocoli-KdpD	0.78
Salmocoli-KdpD	0.83
Streptocoli-KdpD	1.44
Pseudocoli-KdpD	0.35

However production of phosphorylated KdpE should be possible in combination with the likewise decreased kinase-phosphotransferase activities. In summary, replacing the KdpD-Usp domain influences the enzymatic activities of KdpD, explaining altered *kdpFABC *expression patterns in some KdpD chimeras. Importantly, KdpD-UspF and KdpD-UspG are rare examples of KdpD derivatives that lost sensing capabilities *in vivo*, but exhibited kinase, phosphotransferase, and phosphatase activity *in vitro*.

### UspF and UspG differ in surface charge from the *E. coli *KdpD-Usp domain

**Figure 7:**
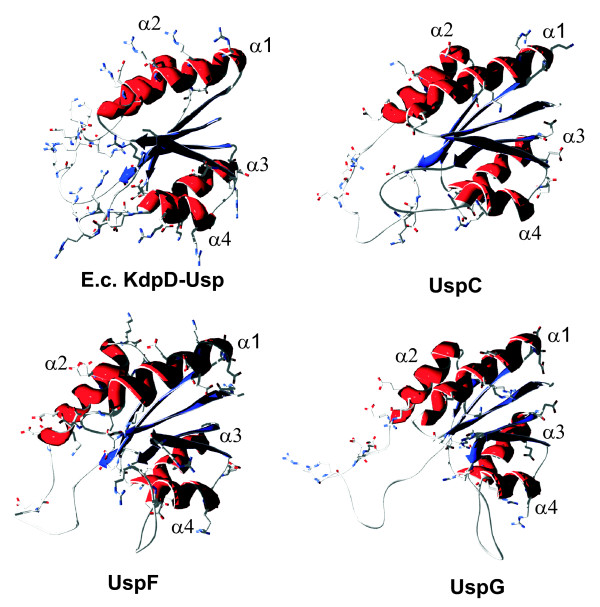
**Surface charge of the Usp domain within KdpD (amino acids 253–373) compared to UspC, UspF, and UspG**. The tertiary structures were obtained by ESyPred3D modeling [29]. All four proteins/protein domains consist of a bundle of four to five β-sheets (blue) surrounded by four α-helices (red). Only charged amino acids are shown. The positively charged side chains are drawn in blue, the negatively charged side chains are drawn in red.

To examine differences between UspF, UspG, UspC and *E. coli *KdpD-Usp, the putative tertiary structures of these proteins/protein domain were generated using ESyPred3D modeling [29]. Although the amino acid sequences of these proteins lack a high degree of sequence identity, all proteins share the same predicted tertiary structure, which consists of a bundle of four to five β-sheets surrounded by four α-helices (Fig. 7). As indicated in Fig. 7, the *E. coli *KdpD-Usp domain is highly charged. The flexible regions between a-helix1 and a-helix2, as well as between β-sheet4 and a-helix4 contain an accumulation of positively charged amino acids (especially Arg), which are not found in UspF or UspG (Fig. 7). In addition, the KdpD-Usp domain contains a cluster of positively charged Arg residues on the surface of a-helix1 (Fig. 7), which are neither present in UspF nor in UspG. In contrast, UspF and UspG are characterized by a predominantly negatively charged surface (Fig. 7). Based on these results, differences in the net surface charges between KdpD-Usp and UspF/UspG may be the reason for the non-functionality of KdpD-UspF and KdpD-UspG *in vivo*. In support of this hypothesis, replacing UspC with the KdpD-Usp domain resulted in a fully functional KdpD. UspC contains a positively charged amino acid cluster between a-helix1 and a-helix2 as well as between β-sheet4 and a-helix5 (Fig. 7).

## Discussion

The N-terminal input domain of the KdpD sensor kinase contains a domain that belongs to the universal stress protein family [18,19]. This domain has been characterized as an interaction site for the soluble UspC protein. Moreover, binding of UspC scaffolds the KdpD/KdpE signaling cascade under salt stress [19]. Since the mechanistic significance of the KdpD-Usp domain for signaling was still unclear, we employed a "domain swapping" approach to replace this domain with four homologous domains or the six soluble Usp proteins. As shown earlier, [19] and corroborated here (Fig. 7), the tertiary structure of all inserted domains is very similar, although the degree of amino acid identity is rather low. In general, we have hypothesized three different mechanisms of how Usp domain swapping could affect KdpD/KdpE signaling: (i) UspC scaffolding under salt stress is increased/abolished due to affinity alterations of the inserted domains towards UspC, (ii) the enzymatic activities of the KdpD chimeras are altered, and (iii) the protein dynamics of the sensor are altered. Interestingly, we generated chimeras covering all these possibilities.

Scaffolding under salt stress was only observed when UspC was inserted into KdpD. In contrast, all other domains prevented scaffolding by UspC. It should be noted that the KdpD-Usp domain sequences differ among bacteria, and also the set of available soluble Usp proteins within these bacteria is variable. *A. tumefaciens *has three *usp *homologues (*atu0496, atu0904*, and *atu1730*), *S. coelicolor *has eleven *usp *homologues (*sco0172, sco0178, sco0167, sco0180, sco0181, sco0198, sco0200, sco0937, sco7156, sco7247*, and *sco7299*), *P. aeruginosa *has seven (*pa1753, pa1789, pa3017, pa3309, pa4328, pa4352*, and *pa5027*), and *S. enterica *serotype Typhimurium has six homologues similar to *E. coli *(*uspA, uspC, uspD, uspE, uspF*, and *uspG*). With the exception of *S. enterica*, none of these organisms has a *uspC *homologue, suggesting that KdpD/KdpE scaffolding either does not exist in these bacteria, or it is mediated by other Usp proteins. This leads to the conclusion that UspC is the specific scaffolding protein for KdpD/KdpE in *E. coli*.

Although all chimeras exhibited enzymatic activity, the ratio between kinase-phosphotransferase to phosphatase activity was shifted in some chimeras. In Pseudocoli-KdpD, the ratio was shifted towards the phosphatase activity, producing a significantly lower expression level than wild-type KdpD. Likewise, KdpD-UspC and Streptocoli-Usp had increased kinase-phosphotransferase to phosphatase ratios and were characterized by significantly higher induction values compared to wild-type KdpD.

**Figure 8:**
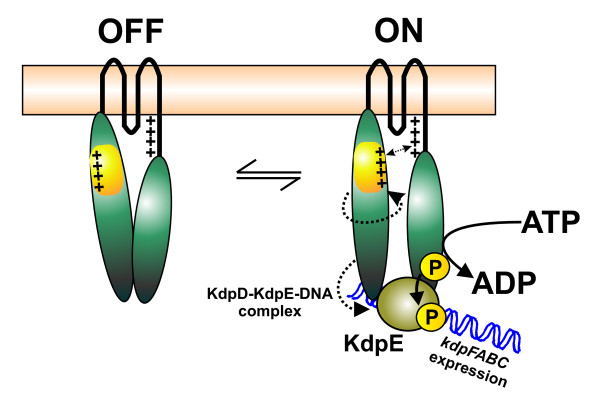
**Model of KdpD activation**. KdpD exists in two states, an "OFF" and an "ON" state. Upon stimulus perception, the positively charged surface of the Usp domain within the N-terminal region (yellow) faces the positively charged region (R503-R511) [10,11,14] in the C-terminal region of KdpD. Thus, electrostatic repulsions between N- and C-terminal domains force the protein into the "open" position. This in turn releases the N-terminal domain, forming a stable complex with KdpE~P and the DNA [25] initiating *kdpFABC *expression. Replacement of the KdpD-Usp domain with UspF or UspG results in inversion of the surface net charges. The negative net surface charge of these two proteins forces electrostatic attraction between the N- and the C-terminal regions, leaving KdpD in the "OFF" state under all conditions.

Last but not least, the "domain swapping" approach identified the first two KdpD derivatives (KdpD-UspG and KdpD-UspF) with alterations in the N-terminal domain that lost the sensing/signal processing (signaling) properties towards K^+ ^limitation, while these proteins exhibited enzymatic activities *in vitro*. The analysis of other chimeras such as KdpD-UspC or KdpD-UspA demonstrates that sensing/signaling was not prevented because of the replacement of the domain *per se*, but that the blockage of the sensor was specifically due to the insertion of UspF or UspG. These data suggest that the N-terminal cytoplasmic domain is important for KdpD/KdpE sensing and/or signaling. We have previously shown that the N-terminal domain of KdpD interacts with the C-terminal region of the protein [25]. Kuhn and coworkers claimed that the C-terminal cytoplasmic domain of KdpD is sufficient to function as a K^+ ^sensor [14]. Indeed, several truncated KdpD derivatives respond to K^+ ^limitation. However in all known examples, these proteins are unable to repress *kdpFABC *at higher external K^+ ^concentrations [14,25]. These data reveal that the N-terminal domain is required for full functionality. Using a comparative analysis of the net surface charges between KdpD-Usp, UspC, UspF, and UspG, we gained new insight on how all these results fit together. In contrast to the highly positively charged surface of the *E. coli *KdpD-Usp domain, UspF and UspG are characterized by a predominantly negatively charged surface. Furthermore, proteins of the UspFG subfamily can be modified by adenylation and phosphorylation [24], which could further enhance the negatively charged surface *in vivo*. Therefore, we propose that alterations in the electrostatic interaction between the large N- and C-terminal domains in KdpD are involved in the activation of the signaling cascade, specifically by autophosphorylation. A previous model suggested that the positioning of the N- and C-terminal domains are critical and probably change upon stimulus perception [8]. It was proposed that the sensor switches from an "OFF" state to an "ON" state [25]. The "ON" state was thought to be achieved by a movement of the two domains towards each other. The charge distribution described here, as well as the activation potential of a sensor that lacks either the N- or C-terminal domain suggests a revision of the former model. The extension of the fourth transmembrane domain located in the C-terminal region of KdpD is characterized by a cluster of positively charged amino acids [10,11]. As the positively charged Usp domain turns towards the C-terminal domain, the protein switches into an open "ON" position by electrostatic repulsion of the positively charged amino acids in the N- and C-terminal domains allowing KdpD/KdpE signaling (Fig. 8). Replacement of the KdpD-Usp domain by the negatively charged UspF and UspG might force the "OFF" state of KdpD due to electrostatic attraction of the N- and C-terminal domains to each other (Fig. 8). A possible explanation why KdpD-UspF and KdpD-UspG are fully active *in vitro *but block *kdpFABC *expression *in vivo *might be that the stabilization of the KdpE-DNA complex by KdpD is prevented in the "OFF" state. This hypothesis is supported by the fact that the separated N-terminal domain (KdpD/1-395) permanently stabilizes the interaction between phosphorylated KdpE and the corresponding DNA-binding site and therefore promotes a constitutive *kdpFABC *expression [25].

## Conclusion

The Usp domain within KdpD is important for proper KdpD/KdpE signaling. Alterations within this domain can completely prevent the response towards K^+ ^limitation as well as salt stress. The KdpD-Usp domain surface contains numerous positively charged amino acids. Electrostatic repulsion and attraction between the N-terminal and C-terminal domain are supposed to be important for KdpD (de)activation. Therefore, the KdpD-Usp domain not only functions as a binding surface for the native scaffold UspC, but also seems to be crucial for internal KdpD signaling, shifting the protein from an "OFF" into an "ON" state.

## Methods

### Materials

[γ^32^-P]ATP and NAP-5 gel filtration columns were purchased from Amersham GE Healthcare. Goat anti-(rabbit IgG)-alkaline phosphatase was purchased from Biomol. All other reagents were reagent grade and obtained from commercial sources.

### Bacterial strains and plasmids

*E. coli *strain JM 109 [*recA1 endA1 gyrA96 thi hsdR17 supE44*λ*relA1 Δ(lac-proAB)/*F'*traD36 proA*^+^*B*^+^*lacI*^*q*^*lacZ*ΔM15] [30] was used as carrier for the plasmids described. *E. coli *strain TKR2000 [Δ*kdpFABCDE trkA405 trkD1 atp706*] [31] containing different variants of plasmid pPV5-3 encoding the different KdpD-Usp derivatives (see below) was used for expression of the *kdp-usp *derivatives from the *tac *promoter. *E. coli *strain HAK006 [Δ*kdpABCD *Δ(*lac-pro*) *ara thi*] [32] carrying a *kdpFABC *promoter/operator-*lacZ *fusion was used to probe signal transduction *in vivo*. *E. coli *LMG194 [F^- ^Δ*lacX74 galE galK thi rpsL ΔphoA *(PvuII) Δ*ara714leu*::Tn*10*] [33] was used for expression of the *kdp-usp *derivatives from the *araBAD *promoter.

To replace the Usp domain in *E. coli *KdpD with the *E. coli *Usp protein sequences, the corresponding *usp *genes were PCR amplified using genomic DNA of *E. coli *MG1655 [34] as a template. The *uspA*, *uspD*, *uspE*, *uspF*, and *uspG *genes were amplified with primers complementary at least 21 bp to the 5' or the 3' ends of the corresponding genes with overhangs for a 5' NsiI site and a 3' SpeI site, respectively. *uspC *was amplified similarly, but with a 5' terminal SacI site. The amplified DNA fragments were cut with NsiI and SpeI, or SacI and SpeI, respectively, and ligated into equally treated vector pPV5-3, resulting in plasmids pPV5-3/UspA, pPV5-3/UspC, pPV5-3/UspD, pPV5-3/UspE, pPV5-3/UspF, and pPV5-3/UspG. To replace the Usp domain of *E. coli *KdpD with the Usp domain of the KdpD proteins of *Agrobacterium tumefaciens*, *Salmonella enterica *serotype Typhimurium, *Streptomyces coelicolor*, and *Pseudomonas aeruginosa*, respectively, the corresponding gene fragments were amplified by PCR using primers which were complementary to the corresponding gene locus with genomic DNA from the abovementioned bacteria as template. The corresponding regions of the *kdpD *gene were amplified with primers complementary at least 21 bp to the 5' or the 3' ends of the corresponding *kdpD *gene locus with overhangs for a 5' SacI site and a 3' SpeI site, respectively. The amplified DNA fragments were cut with SacI and SpeI, respectively, and ligated into equally treated vector pPV5-3, resulting in plasmids pPV5-3/Agrocoli-KdpD, pPV5-3/Salmocoli-KdpD, pPV5-3/Streptocoli-KdpD, and pPV5-3/Pseudocoli-KdpD. All hybrid genes were verified by sequencing each PCR-generated DNA segment through the ligation junctions in double-stranded plasmid DNA. The *kdpD *derivatives *kdpD-uspA*, *kdpD-uspD*, *kdpD-uspE*, *kdpD-uspG*, *kdpD-uspF*, *agrocoli-kdpD*, *salmocoli-kdpD*, and *pseudocoli-kdpD *were cloned into plasmid pBAD-18 [33] using XmaI and HindIII; *kdpD-uspC *and *pseudocoli-kdpD *were cloned into plasmid pBD (*kdpD *in pBAD-18) [35] using XhoI and SpeI resulting in plasmids pBD/UspA, pBD/UspC, pBD/UspD, pBD/UspE, pBD/UspF, pBD/UspG, pBD/Agrocoli-KdpD, pBD/Salmocoli-KdpD, pBD/Streptocoli-KdpD, and pBD/Pseudocoli-KdpD, respectively. The correct insertion of the respective *kdpD *derivatives was checked by restriction analysis of the corresponding plasmids.

### Cell fractionation and preparation of inverted membrane vesicles

*E. coli *strain TKR2000 transformed with plasmids pPV5-3 or its derivatives carrying different *kdpD*-*usp *derivatives was grown aerobically at 37°C in KML complex medium (1% tryptone, 0.5% yeast extract, and 1% KCl) supplemented with ampicillin (100 μg/ml). Cells were harvested at an absorbance at 600 nm of ~1.0, washed with buffer (50 mM Tris/HCl pH 7.5, 10 mM MgCl_2_) and disrupted by passage through a Cell disruptor (Constant Cell Disruption Systems, Northants, UK) at 1.35 kbar and 4°C in disruption buffer [50 mM Tris/HCl pH 7.5, 10% (v/v) glycerol, 10 mM MgCl_2_, 1 mM dithiotreitol, 0.5 mM phenylmethylsulfonylfluoride, and 0.03 mg/ml (w/v) DNAse]. After removal of intact cells and cell debris by centrifugation (9.000 × g, 10 min), membrane vesicles were collected by centrifugation at 160.000 × g for 60 min. Membrane vesicles were washed with low ionic strength buffer (10 mM Tris/HCl, pH 7.5, 3 mM EDTA), centrifuged again and resuspended in 50 mM Tris/HCl, pH 7.5 containing 10% (v/v) glycerol. Vesicles were frozen in liquid nitrogen and stored at -80°C until use.

### Phosphorylation and Dephosphorylation Assays

Inverted membrane vesicles (2 mg protein/ml) containing about 10% KdpD were incubated at room temperature in phosphorylation buffer [50 mM Tris/HCl, pH 7.5, 10% glycerol (v/v), 0.5 M NaCl, 10 mM MgCl_2 _and 2 mM DTT]. Phosphorylation was initiated by addition of 20 μM [γ-^32^P]ATP (2.38 Ci/mmol). At different times, aliquots were removed and the reaction was stopped by mixing with SDS-sample buffer [36]. After incubation for 4.5 min, an equimolar amount of purified KdpE was added to the KdpD-containing samples and the incubation was continued. Further aliquots were removed at different times and mixed with SDS-sample buffer [36]. For dephosphorylation assays, 10His-KdpE~^32^P was obtained as described [16,37]. Dephosphorylation was initiated by addition of inverted membrane vesicles (1 mg/ml) containing KdpD or KdpD chimeras, 20 mM MgCl_2 _in presence and absence of 20 μM ATP-γ-S. At different times, aliquots were removed, and the reaction was stopped by addition of SDS-sample buffer. All samples were immediately subjected to SDS-polyacrylamide gel electrophoresis

PAGE, an [γ-^32^P]ATP standard was loaded on the gels. Gels were dried, and protein phosphorylation was detected by exposure of the gels to a Storage Phosphor Screen. Phosphorylated proteins were quantified by image analysis using the Phosphorimager Storm (GE Healthcare).

### Determination of *kdpFABC *expression *in vivo*

*In vivo *signal transduction was probed using *E. coli *strain HAK006 transformed with the plasmids as previously described. Cells were grown in minimal media containing different concentrations of K^+ ^[38] or in minimal medium containing 5 mM K^+ ^with or without 0.4 M sodium chloride, and harvested in the mid-exponential growth phase by centrifugation. β-galactosidase activity was determined as described [39] and is given in Miller Units.

### Analytical Procedures

Proteins were assayed using a modified Lowry method [40], using bovine serum albumin as a standard. Immunodetection of KdpD was performed with polyclonal antibodies against KdpD as previously described [41].

### Sequence Comparisons

Amino acid sequences were compared using the VectorNTI alignment tool AlignX (Invitrogen, Karlsruhe, Germany). Structure predictions were performed by ESyPred3D modeling [29] on the expasy server http://www.expasy.ch.

## Authors' contributions

RH and KJ designed research experiments; ML constructed the *kdpD*-hybrid genes; RH and ML performed experiments and analyzed data. KJ and RH wrote the manuscript. All authors have read and approved the final manuscript.
